# Immunohistochemical Analysis of Neurotransmitters in Neurosecretory Protein GL-Producing Neurons of the Mouse Hypothalamus

**DOI:** 10.3390/biomedicines10020454

**Published:** 2022-02-15

**Authors:** Mana Naito, Eiko Iwakoshi-Ukena, Shogo Moriwaki, Yuki Narimatsu, Masaki Kato, Megumi Furumitsu, Yuta Miyamoto, Shigeyuki Esumi, Kazuyoshi Ukena

**Affiliations:** 1 Laboratory of Neurometabolism, Graduate School of Integrated Sciences for Life, Hiroshima University, Hiroshima 739-8521, Japan; m205336@hiroshima-u.ac.jp (M.N.); iwakoshi@hiroshima-u.ac.jp (E.I.-U.); m203300@hiroshima-u.ac.jp (S.M.); d214243@hiroshima-u.ac.jp (Y.N.); d214882@hiroshima-u.ac.jp (M.K.); mfurumi@hiroshima-u.ac.jp (M.F.); 2 Department of Anatomy and Neurobiology, Graduate School of Medical Sciences, Kumamoto University, Kumamoto 860-8556, Japan; ymiyamoto@kumamoto-u.ac.jp (Y.M.); esumi@kumamoto-u.ac.jp (S.E.)

**Keywords:** neurotransmitter, GABA, catecholamine, glutamate, neurosecretory protein GL, hypothalamus, colocalization, immunohistochemistry, feeding behavior, central nervous system

## Abstract

We recently discovered a novel neuropeptide of 80 amino acid residues: neurosecretory protein GL (NPGL), in the hypothalamus of birds and rodents. NPGL is localized in the lateral posterior part of the arcuate nucleus (ArcLP), and it enhances feeding behavior and fat accumulation in mice. Various neurotransmitters, such as catecholamine, glutamate, and γ-aminobutyric acid (GABA), produced in the hypothalamus are also involved in energy metabolism. The colocalization of neurotransmitters and NPGL in neurons of the ArcLP leads to the elucidation of the regulatory mechanism of NPGL neurons. In this study, we performed double immunofluorescence staining to elucidate the relationship between NPGL and neurotransmitters in mice. The present study revealed that NPGL neurons did not co-express tyrosine hydroxylase as a marker of catecholaminergic neurons and vesicular glutamate transporter-2 as a marker of glutamatergic neurons. In contrast, NPGL neurons co-produced glutamate decarboxylase 67, a marker for GABAergic neurons. In addition, approximately 50% of NPGL neurons were identical to GABAergic neurons. These results suggest that some functions of NPGL neurons may be related to those of GABA. This study provides insights into the neural network of NPGL neurons that regulate energy homeostasis, including feeding behavior and fat accumulation.

## 1. Introduction

The hypothalamus is an important part of the brain and controls basic physiological functions, including energy homeostasis, fluid/electrolyte balance, thermoregulation, stress responses, growth, and reproductive behaviors [1]. Among them, feeding behavior is mainly regulated by various feeding-related factors in the hypothalamus [2]. For instance, neuropeptide Y (NPY) and agouti-related peptide (AgRP) enhance feeding behavior, whereas α-melanocyte-stimulating hormone (α-MSH) derived from pro-opiomelanocortin (POMC) and cocaine- and amphetamine-regulated transcript (CART) inhibit feeding behavior [3,4,5]. 

These neuropeptides are produced in the arcuate nucleus (Arc) [3,4,5]. Melanin-concentrating hormone (MCH) and orexin, which are expressed in the lateral hypothalamus area (LHA), are known orexigenic factors [6,7]. Feeding behavior is also regulated by factors produced in the peripheral tissues. 

Cholecystokinin (CCK) and glucagon-like peptide 1 (GLP-1) are anorexigenic hormones secreted from the gut [8,9,10]. Peptide YY_3–36_ (PYY_3–36_) is also secreted from the gut and acts on the hypothalamus [11,12,13]. PYY_3–36_ inhibits feeding behavior by attenuating NPY/AgRP neurons [12]. Ghrelin, secreted from the stomach, enhances appetite by activating NPY/AgRP neurons and inhibiting POMC neurons in the hypothalamus [14,15]. Leptin, an anorexigenic adipocytokine secreted from white adipose tissue, influences NPY/AgRP and POMC neuron activities [16,17,18]. Moreover, insulin, a pancreatic hormone, maintains systemic glucose homeostasis and inhibits feeding behavior via the hypothalamus [19,20].

Neurotransmitters are also involved in the regulation of feeding behavior [6,21]. Serotonin suppresses appetite [2]. Dopamine attenuates appetite in the Arc and the LHA, whereas it increases appetite in the ventromedial hypothalamus (VMH) [22,23,24]. Glutamate participates in the regulation of feeding behavior [21]. In addition, γ-aminobutyric acid (GABA), an inhibitory neurotransmitter produced by NPY/AgRP neurons, enhances feeding behavior by inhibiting anorexigenic neurons, such as POMC neurons [25,26]. Thus, feeding behavior is intricately modulated by various neurotransmitters and neuropeptides. However, this complex system remains to be fully elucidated. To further understand the mechanisms of feeding behavior and energy homeostasis, we sought to identify previously unknown hypothalamic neuropeptides in vertebrates.

Recently, we discovered a novel gene related to feeding behavior and energy homeostasis in the chicken hypothalamus [27]. The small secretory protein derived from this novel gene was named neurosecretory protein GL (NPGL) because the C-terminal amino acid sequence is Gly-Leu-NH_2_ [27]. Genome database analysis suggested that the homologous gene of NPGL is conserved in vertebrates, including chickens, mice, rats, and humans [27,28,29]. 

Functional analysis of NPGL has been conducted in mammalian models, such as mice and rats. *Npgl* mRNA expression is increased by fasting in mice, whereas it is reduced by a high-fat diet [29]. In addition, the administration of NPGL and *Npgl* overexpression in the hypothalamus increases food intake and fat accumulation in rats and mice [28,30,31]. These data suggest that NPGL is involved in the energy metabolism of rodents.

In a previous study, we found that NPGL-producing cell bodies are localized in the lateral posterior part of the Arc (ArcLP), which is involved in feeding regulation, and fibers of NPGL neurons project to several hypothalamic regions, including POMC neurons in mice [29]. Furthermore, some NPGL neurons co-produce the orexigenic neuropeptide galanin in mice [31]. However, the production of neurotransmitters by NPGL-producing neurons remains unknown. 

The information will provide vital knowledge to clarify the regulatory mechanisms of NPGL neurons on energy metabolism. The aim of this study was to elucidate the neural characteristics of NPGL neurons. In this study, we performed double immunofluorescence staining to investigate the relationship between NPGL and neurotransmitters, that is, catecholamine, glutamate, and GABA, in the mouse hypothalamus using antibodies against marker proteins of neurotransmitters.

## 2. Materials and Methods

### 2.1. Animals

C57BL/6J mice (*n* = 4–6 in each double immunofluorescence staining) were purchased from Nihon SLC (Shizuoka, Japan) and singly housed under standard conditions (25 ± 2 °C under a 12-h light/12-h dark cycle) with ad libitum access to water and normal chow (CE-2; CLEA Japan, Tokyo, Japan). Animal surgery was performed under isoflurane anesthesia. All animal experiments were performed in accordance with the Guide for the Care and Use of Laboratory Animals, prepared by Hiroshima University (Higashi-Hiroshima, Japan), and these procedures were approved by the Institutional Animal Care and Use Committee of Hiroshima University (permit number: C19-18).

### 2.2. Immunohistochemistry

In stereotaxic surgery, male mice (8 weeks old) were injected with colchicine (30 μg/2.5 μL) into the lateral ventricle under isoflurane anesthesia. The colchicine treatment enabled the detection of NPGL-immunoreactive cell bodies [29]. After 2 days of colchicine treatment, the brains were cut into 20- or 40-µm sections with a cryostat at –20 °C. The procedure using immunofluorescence staining of the floating sections was conducted as previously described [29,31].

The co-expression of NPGL-immunoreactive cell bodies with neurotransmitters was surveyed using double-label immunofluorescence as follows: a guinea pig antibody against NPGL (1:500 dilution) and a rabbit antibody against tyrosine hydroxylase (TH), as a marker of catecholaminergic neurons (1:2000 dilution, AB152; Merck Millipore, Burlington, MA, USA), were used for the detection of NPGL neurons. For catecholaminergic neurons, a rabbit antibody against NPGL (1:250 dilution), and a guinea pig antibody against vesicular glutamate transporter 2 (vGLUT2), as a marker of glutamatergic neurons (1:1000 dilution, RRID_2571621; Frontier Institute Co., Ltd, Hokkaido, Japan), were used for the detection of NPGL neurons and glutamatergic neurons. 

A guinea pig antibody against NPGL (1:500 dilution) and a rabbit antibody against glutamic acid decarboxylase (GAD67), as a marker of GABAergic neurons (1:2000 dilution, GTX113190; GeneTex, Inc., Irvine, CA, USA), were used for the detection of NPGL neurons and GABAergic neurons. Alexa Fluor 568-conjugated donkey anti-rabbit IgG (1:500 dilution; ab175470; Abcam, Cambridge, UK), Cy3-conjugated donkey anti-rabbit IgG (1:400 dilution; 711-165-152; Jackson ImmunoResearch Laboratories, West Grove, PA, USA), and Alexa Fluor 488-conjugated donkey anti-guinea pig IgG (1:600 dilution; 706-545-148; Jackson ImmunoResearch Laboratories) were used as secondary antibodies. Immunoreactive labeling was observed using an Eclipse E600 conventional microscope (Nikon, Tokyo, Japan) or an FV3000 confocal microscope (Olympus, Tokyo, Japan).

### 2.3. NPGL-Immunoreactive Cells Co-Expressing with GAD67 Counting

For quantitative analysis of NPGL-immunoreactive cells co-expressing GAD67, images of all localized NPGL-immunoreactive cells were photographed. Based on the observed images, the total number of NPGL-immunoreactive cells was counted at 20 µm intervals, that is, skipping one slice. Next, the number of NPGL-immunoreactive cells co-expressing GAD67 was counted, and the ratio of the number of NPGL-immunoreactive cells co-expressing GAD67 to the total number of NPGL-immunoreactive cells was calculated. All results are presented as the mean ± standard error of the mean (SEM, *n* = 6).

## 3. Results

### 3.1. Double Immunofluorescence Staining of NPGL and TH as a Marker of Catecholaminergic Neurons

**Figure 1:**
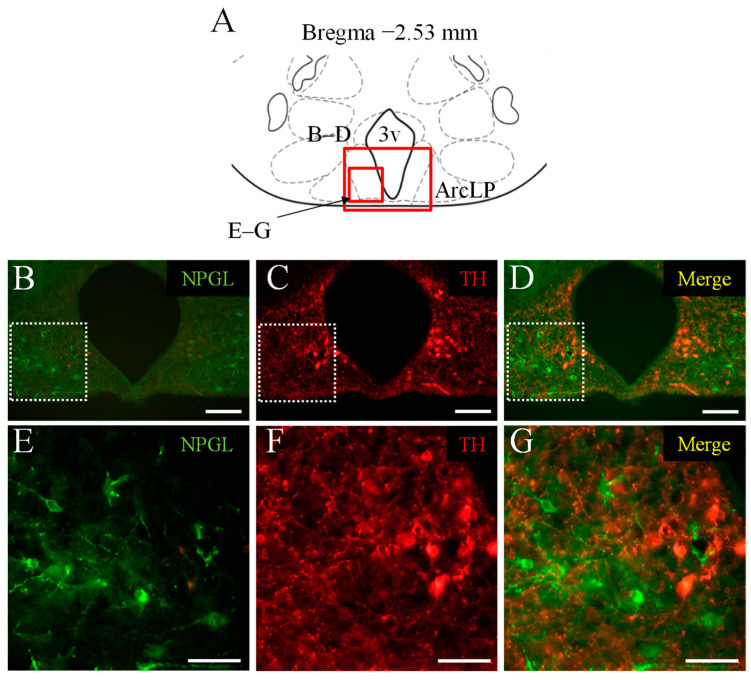
Localization of NPGL-producing neurons and catecholaminergic neurons. The regions in the photomicrographs (**B**–**G**) depicted in the schematic illustration (**A**) are shown as red solid boxes. The distribution of NPGL-immunoreactive cells and tyrosine hydroxylase (TH)-immunoreactive cells in the lateral posterior part of the arcuate nucleus (ArcLP) near the third ventricle (3v) and other hypothalamus regions using the conventional microscope. The dotted squares in (**B**–**D**) are shown magnified in (**E**–**G**). Scale bars = 100 µm (**B**–**D**) and 50 µm (**E**–**G**).

To examine the co-expression of NPGL and catecholamine in the hypothalamus, double immunofluorescence staining was performed using antibodies against NPGL and TH. NPGL-immunoreactive cells were localized in the ArcLP (Figure 1B,E). TH-immunoreactive cells were also observed in the neighborhood of NPGL-producing cells in the ArcLP (Figure 1C,F), in addition to other regions of the ArcLP (Figure 1C). The merged image showed that NPGL-immunoreactive cells were different from TH-positive neurons, that is, catecholaminergic neurons (Figure 1D,G). Similar results were obtained in repeated experiments with four mice.

### 3.2. Double Immunofluorescence Staining of NPGL and vGLUT2 as a Marker of Glutamatergic Neurons

**Figure 2:**
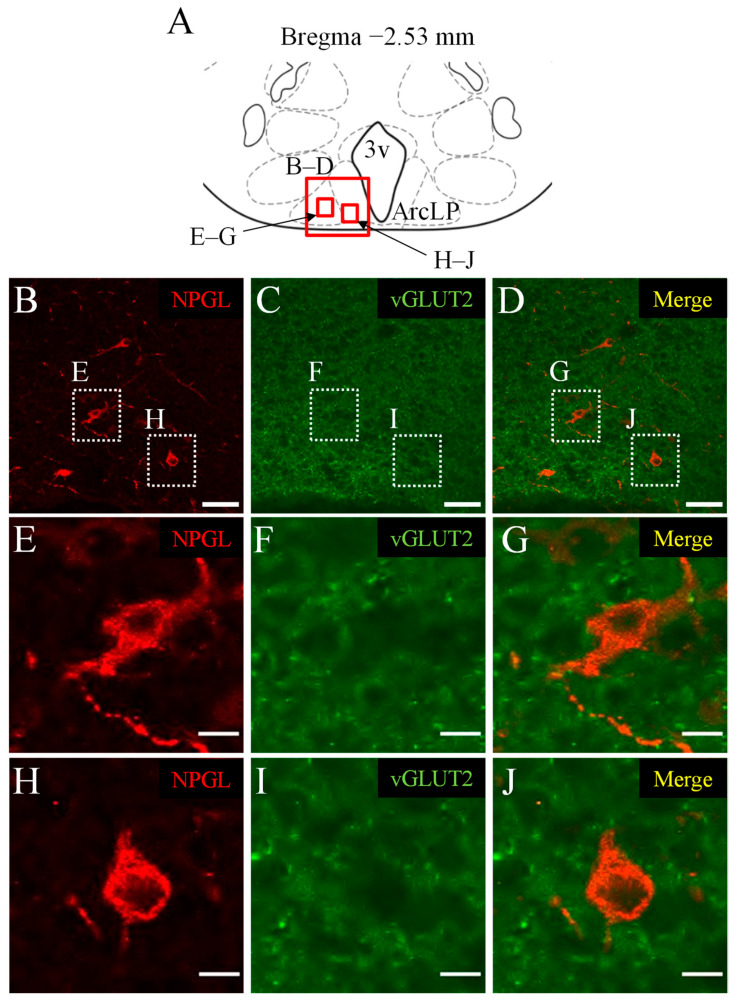
Localization of NPGL-producing neurons and glutamatergic neurons. The regions in the photomicrographs (**B**–**J**) depicted in the schematic illustration (**A**) are shown as red solid boxes. The distribution of NPGL-immunoreactive cells and vesicular glutamate transporter-2 (vGLUT2)-immunoreactive reactions in the lateral posterior part of the arcuate nucleus (ArcLP) near the third ventricle (3v) using the confocal microscope. vGLUT2-immunoreactive reactions were observed in granular form (**C**,**F**,**I**). The dotted squares in (**B**–**D**) are shown magnified in (**E**–**J**). Scale bars = 50 µm (**B**–**D**) and 10 µm (**E**–**J**).

Next, we investigated the colocalization of NPGL and glutamatergic neurons using an antibody against vGLUT2. NPGL-immunoreactive cells were localized in the ArcLP (Figure 2B,E,H). vGLUT2-immunoreactivities were observed in the granular form in the hypothalamus (Figure 2C,F,I). The merged images showed that NPGL-immunoreactive cells did not contain vGLUT2-immunoreactive granules (Figure 2D,G,J). Similar results were obtained in repeated experiments with four mice.

### 3.3. Double Immunofluorescence Staining of NPGL and GAD67 as a Marker of GABAergic Neurons

**Figure 3:**
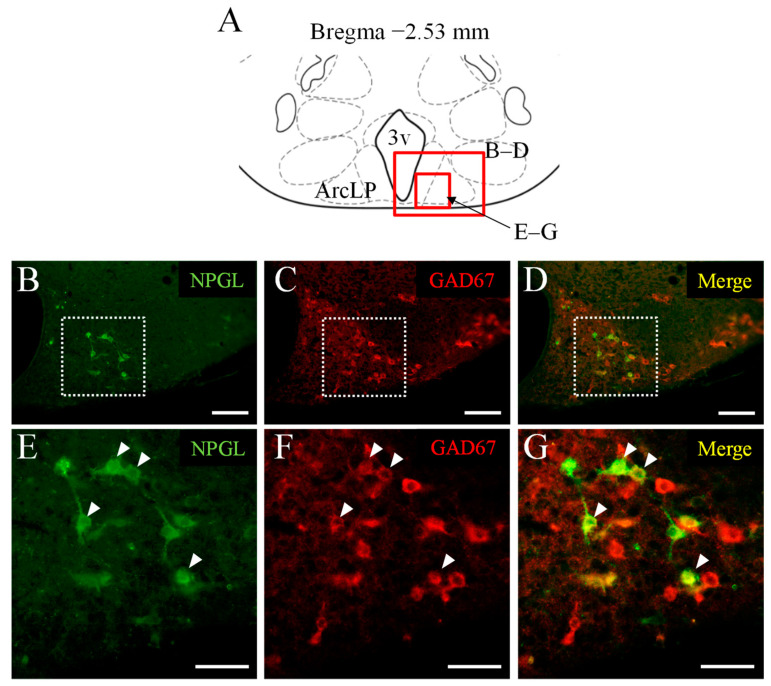
Localization of NPGL-producing neurons and GABAergic neurons. The regions in the photomicrographs (**B**–**G**) depicted in the schematic illustration (**A**) are shown as red solid boxes. The distribution of NPGL-immunoreactive cells and glutamate decarboxylase 67 (GAD67)-immunoreactive cells in the lateral posterior part of the arcuate nucleus (ArcLP) near the third ventricle (3v) and other hypothalamus regions using the conventional microscope. The dotted squares in (**B**–**D**) are shown magnified in (**E**–**G**). Arrowheads show co-expressed NPGL-immunoreactive cells with GAD67. Scale bars = 100 µm (**B**–**D**) and 50 µm (**E**–**G**).

In the final experiment, we analyzed the colocalization of NPGL and GABAergic neurons using an antibody against GAD67. NPGL-immunoreactive cells were localized in the ArcLP (Figure 3B,E). GAD67-immunoreactive cells were also localized in the ArcLP, in addition to other hypothalamic regions (Figure 3C,F). The merged images indicated that NPGL was co-expressed with GAD67 in some NPGL-immunoreactive cells (Figure 3D,G). Similar results were obtained in repeated experiments with six mice. The number of NPGL-immunoreactive cells expressing GAD67 was counted. Based on the estimated number of cells, approximately 50% of NPGL cells were co-produced with GAD67, showing GABAergic neurons (Table 1).

## 4. Discussion

Our previous studies have indicated that NPGL participates in energy homeostasis, including feeding behavior and fat accumulation in rodents [28,29,30,31]. In addition, we found that NPGL cell bodies are localized in the ArcLP, and NPGL-containing fibers project to several anterior hypothalamic regions in mice [29]. However, the colocalization of neurotransmitters in NPGL-producing neurons remains unknown. In this study, we used an immunohistochemical analysis to investigate whether NPGL neurons produce other neurotransmitters in the mouse hypothalamus. The data showed that a subset of NPGL-producing neurons were GABAergic. These results suggest that GABA is also involved in the neural network and/or function of NPGL neurons.

Catecholamines, including adrenaline, noradrenaline, and dopamine, are excitatory neurotransmitters. In rodents, the injection of noradrenaline or dopamine into the hypothalamus stimulates and inhibits feeding behavior, respectively [2,32]. Glutamate is an excitatory neurotransmitter [21]. In the hypothalamus, some glutamatergic neurons participate in the regulation of feeding behavior [21,33]. However, the present study revealed that NPGL-producing cells did not co-produce TH and vGLUT2. Hence, NPGL neurons are not identical to the catecholaminergic and glutamatergic neurons.

GABA is an inhibitory neurotransmitter in adult mammals [21,26]. In the hypothalamus, GABA is involved in feeding regulation [26]. In the Arc, GABA produced in NPY/AgRP neurons is orexigenic [21,26,34]. In contrast, approximately half of anorexigenic POMC neurons are GABAergic neurons that project to the dorsomedial hypothalamus (DMH) [35]. MCH-expressing neurons containing GABA in the LHA project to several parts of the hypothalamus, and MCH enhances feeding behavior via GABA [26]. In the DMH, GABAergic neurons project to the paraventricular nucleus of the hypothalamus (PVH) and stimulate feeding behavior [36]. 

Taken together, these results show that GABA is an important factor in regulating feeding behaviors in both stimulatory and inhibitory manners in the different hypothalamic regions. In the present study, approximately 50% of NPGL neurons were identical to GABAergic neurons. Previous studies have revealed that fibers of NPGL neurons in the ArcLP innervate POMC neurons in the Arc [29]. Some orexigenic NPY/AgRP neurons exert hyperphagic effects by inhibiting POMC neurons via GABA [25]. 

Therefore, similar to NPY/AgRP neurons, NPGL may exert its hyperphagic effects by inhibiting POMC neurons via GABA. However, it is unclear whether NPGL neurons containing GABA project to the POMC neurons. In the future, it will be necessary to elucidate whether NPGL neurons innervate POMC neurons with or without GABA using retrograde tracers. This type of study will reveal the connection between NPGL/GABA, NPGL, and POMC neurons.

In addition to the cell bodies of NPGL in the ArcLP, fibers of NPGL neurons spread to several areas of the hypothalamus, including the Arc, PVH, DMH, VMH, and LHA [29]. There are some anorexigenic factors, such as corticotropin-releasing hormone (CRH) in the PVH and pituitary adenylate cyclase-activating polypeptide (PACAP), brain-derived neurotrophic factor (BDNF), and steroidogenic factor 1 (SF-1) in the VMH [37,38,39,40]. Furthermore, it has been reported that endogenous bioactive peptides, such as kisspeptin and hemopressin as well as exogenous peptides derived from food proteins, participate in appetite control through the orexigenic and anorexigenic factors as well as the receptors for neurotransmitters or cannabinoids [41,42,43]. 

Therefore, NPGL neurons may influence these various factors or receptors to elicit some important biological functions, including energy homeostasis, feeding behavior, and instinctive behavior. Information on the receptor for NPGL is essential to elucidate the target sites of NPGL. However, a receptor for NPGL has not yet been identified. On the other hand, much attention has been focused on the relationship between eating disorder and dysfunction of neuropeptides [44]. 

Several synthetic bioactive peptides related to urocortin 3, growth hormone-releasing hormone, neuromedin U, and kisspeptin, have antidepressant-like or anxiety-like effects via the receptors for neurotransmitters or the release of neurotransmitters [45,46,47,48]. The dysregulations of neurotransmitters, including GABA and glutamate cause mood disorders and Alzheimer’s disease [49,50]. Taken together, further studies of NPGL may uncover the mechanisms of eating disorders, including anorexia nervosa and brain dysfunctions.

In the present study, approximately 50% of NPGL-producing cells did not contain GABA. About half of the POMC neurons are also GABAergic; however, some POMC neurons express vGLUT2, a marker of glutamatergic neurons [35]. These data suggest that different subpopulations of POMC neurons play diverse physiological roles [35]. Therefore, it is likely that NPGL-producing neurons, like POMC neurons, have multiple neuronal networks and physiological functions. 

Previous studies have revealed that NPGL is co-expressed with galanin, which exerts feeding behavior [51], in approximately 30% of NPGL-producing cells in mice [31]. In addition, some galanin-producing neurons are GABAergic [52,53]. Therefore, it is necessary to analyze whether galanin neurons containing GABA are co-expressed with NPGL using triple-immunostaining for NPGL, GABA, and galanin in future studies. Hence, NPGL-producing cells may have heterogeneities and/or subclasses based on neurotransmitter and neuropeptide phenomena. 

In the future, to clarify the more complex characteristics of NPGL-producing cells containing GABA and other neuropeptides, single-cell transcriptional analysis of NPGL neurons using RNA-Seq appears to be beneficial for the identification of specific transcriptions, including neurotransmitters, neuropeptides, and receptors. A recent single-cell analysis revealed functional divergence and neuronal networks of neurons involved in feeding- and reward-related behaviors [54].

This is the first report of a subset of GABAergic NPGL neurons in the vertebrate hypothalamus. This study will help us understand the neural circuit and functions of NPGL as well as the detailed mechanisms of regulation of energy homeostasis, including feeding behavior and fat accumulation in animals.

## Figures and Tables

**Table 1:** The numbers and ratio of NPGL-immunoreactive cells containing glutamate decarboxylase 67 (GAD67)-immunoreactivities. *n* = 6.

NPGL-immunoreactive cells	373 ± 22.1
NPGL-immunoreactive cells containing GAD67 (No.)	188 ± 18.2
NPGL-immunoreactive cells containing GAD67 (%)	50.1 ± 3.1

## Data Availability

The raw data supporting the findings of this study will be made available by the corresponding author, K.U., to any qualified researchers upon reasonable request.
